# Supplementary material for: Neuromorphic photonic computing with an electro-optic analog memory
Source: Nat Commun. 2026 Feb 7;17:2472. doi: 10.1038/s41467-026-69084-x (PMC12992803; doi:10.1038/s41467-026-69084-x)
Supplement: Supplementary file 1 — Supplementary Information [file 41467_2026_69084_MOESM1_ESM.pdf]

Supplementary: Neuromorphic Photonic Computing with an Electro-Optic Analog Memory

Sean Lam[1*], Ahmed Khaled[2], Simon Bilodeau[3], Bicky A. Marquez[2], Paul R. Prucnal[3], Lukas Chrostowski[1], Bhavin J. Shastri[2,3,4*], Sudip Shekhar[1*]

[1]Electrical and Computer Engineering, University of British Columbia, 5500 – 2332 Main Mall, Vancouver, V6T 1Z4, British Columbia, Canada.
[2]Centre for Nanophotonics, Physics, Engineering Physics & Astronomy, Queen's University, 64 Bader Lane, Kingston, K7L 3N6, Ontario, Canada.
[3]Electrical and Computer Engineering, Princeton University, 41 Olden Street, Princeton, 08544, New Jersey, United States.
[4]Smith Engineering, Electrical and Computer Engineering, Queen's University, 19 Union Street, Kingston, K7L 3N6, Ontario, Canada.

*Corresponding author(s). E-mail(s): seanlm@student.ubc.ca; shastri@ieee.org; sudip@ece.ubc.ca;
Contributing authors: 20ak41@queensu.ca; sbilodeau@princeton.edu; bama@queensu.ca; prucnal@princeton.edu; lukasc@ece.ubc.ca;

1 Supplementary Note 1. Optical Characteristics

From the static optical characteristics of the MRRs in Supplementary Fig. 1a and 1b, the minimum wavelength spacing between different rings is 0.34 nm, the free spectral range (FSR) is 4.67 nm, the extinction ratio is between 15.6 dB and 19.6 dB, and the quality factor is about 50,000. From Supplementary Fig. 1a, optical bus power is estimated to be -6 dBm. In Supplementary Fig. 1c and Supplementary Fig. 1d, to observe the tuning efficiency of the PN junction MRRs, one MRR (peak 3) is biased at various voltages from 0 to 3 V (3 V is the limit for the thick oxide transistors) while the other MRRs (peaks 1, 2, and 4) are biased at zero voltage. Several spectra measurements are conducted at each tuned voltage where the resonance wavelengths are extracted. With multiple measurements at the same tuned voltage, the resonance wavelengths are averaged. Therefore, peaks 1, 2, and 4 reveal system level deviation from a nominal resonance wavelength, due to thermal variations from measurement to measurement, despite using a temperature controller for the chip. Peak 3 shifts to longer wavelengths relative to the other peaks because the increasing reverse bias voltages on this MRR cause a plasma dispersion effect to shift the resonance wavelength. The resonance wavelength and optical power of each MRR are observed at each voltage bias point to track tuning. Supplementary Fig. 1c reveals that as peak 3 is biased, peaks 1, 2, and 4 track similar trajectories, meaning optical crosstalk between adjacent resonances is negligible. In Supplementary Fig. 1d, the resonance power shift is less apparent than the resonance wavelength shift because the power shift occurs near the resonance of the ring, where the ring response's slope is shallow. Looking at the tuning efficiency of the MRR in Supplementary Fig. 1e and 1f, the wavelength and power shifts are 6.22 pm/V and 0.23 dB/V, respectively.

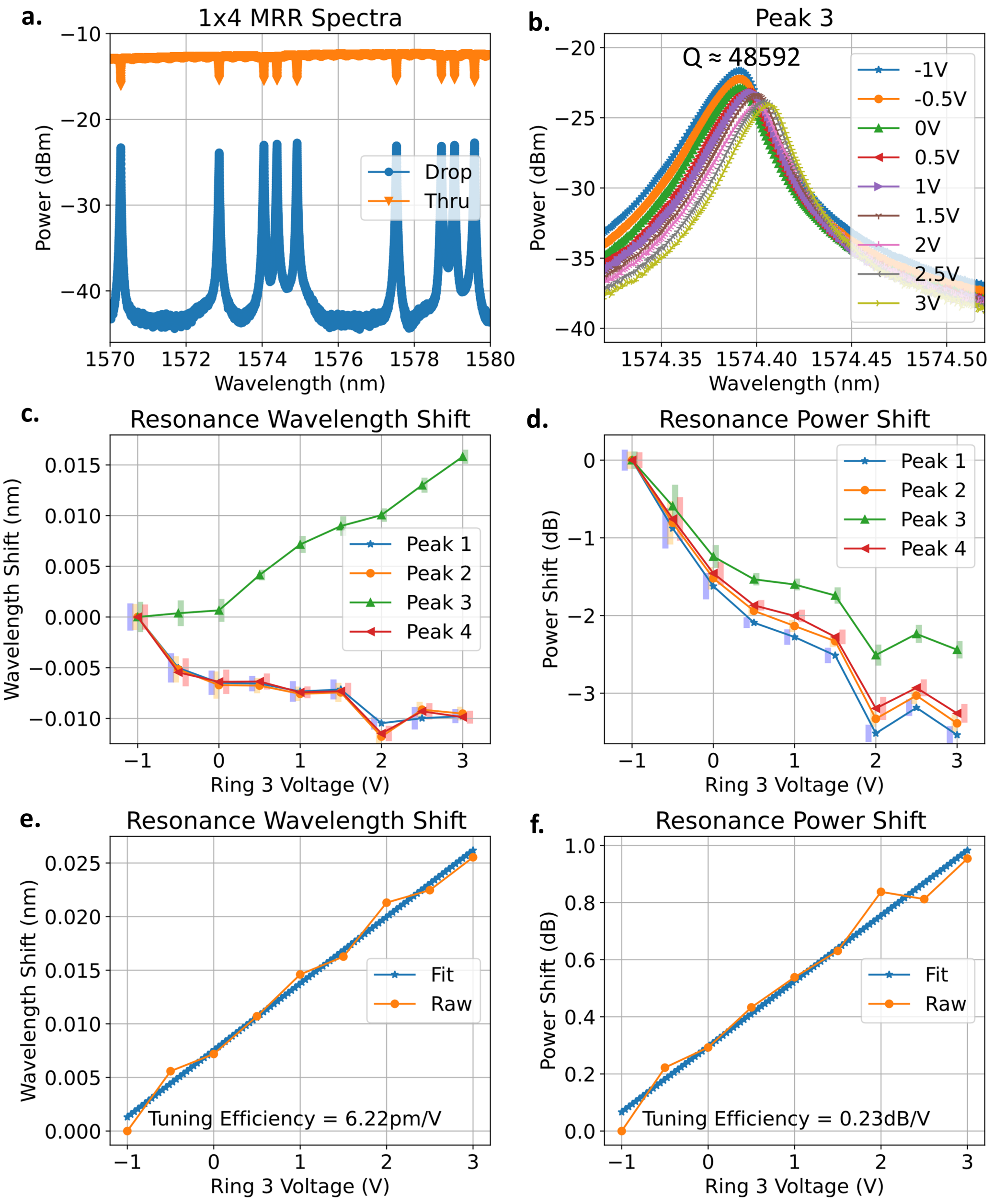

Supplementary Fig. 1: Measured optical characteristics of the neuromorphic photonic weight bank with -6 dBm optical bus power. a) 1x4 MRR spectra showing minimum and maximum drop port extinction ratios of 15.6 dB and 19.9 dB, respectively, and a minimum resonance spacing of 0.34 nm. b) Ring resonance Q factor measures around 50k and the static voltage tuning of resonance is shown. Resonance wavelength shift in c) and power shift in d) is tracked on ring 3 as its bias is swept from -1 V to 3 V, while the other rings remain biased at -1 V. Error bars represent the standard deviation in the wavelength shift and power shift. e) Resonance wavelength shift in e) and power shift in f) using the other three MRR responses to calibrate for effective shifts.

2 Supplementary Note 2. Electro-Optic Characteristics

The leakage is measured with optical bus powers swept from -7.5 dBm to 0.5 dBm. Optical bus power represents the optical power in the bus waveguide that couples light into the MRRs. The leakage depends on the optical power in the circuit. At 0 V, the laser wavelength is biased on the MRR's resonance, meaning the highest optical power is incident on the PN junction in the MRR and the highest leakage is observed. As the SMU sweeps the voltage on the analog memory and PN junction MRR to 2 V, the MRR's resonance shifts away from the laser wavelength, meaning less optical power is circulating in the MRR, less optical power is incident on the PN junction, and less leakage is observed. At 0.5 dBm optical bus power in the circuit, leakage is as high as 200 nA, but at -7.5 dBm optical bus power in the circuit, the leakage is a few nanoamperes. With high optical power incident on the PN junction, additional carriers are generated and are measured as additional leakage. As the voltage bias increases, the MRR resonance strays away from the laser wavelength and less optical power is inside the MRR, meaning less carriers are generated and less leakage is measured. At lower optical powers, such as -7.5 dBm, a couple orders of magnitude reduction in leakage is attained in comparison to 0.5 dBm optical bus power, meaning the retention time can be improved by using lower optical power.

The maximum weight retention time is crucial to revealing the limit in multiply-accumulate (MAC) operations per second in a neural network. The amount of MAC operations per second is dependent on the input data modulation speed, compute time, and weight retention time. Increasing the input data modulation speed and decreasing compute time increases the amount of MAC operations per second, but if the weight retention time is short, inaccurate inferences can occur, meaning that the amount of useful MAC operations is limited by the weight retention time. The maximum retention time is 0.8345 ms for one time constant with 0.5 dBm optical bus power.

Combining data from retention time and leakage, the amount of energy stored in the analog memory capacitor can be estimated. Although the retention time measurement includes nonlinearity from the MRR Lorentzian response, an approximation of the energy consumed by the capacitors provides a baseline to compare with other analog memory technologies. To estimate the energy consumed from leakage, the maximum and minimum PD voltage in the retention time measurement correspond to the minimum and maximum leakage values at 2 V and 0 V on the capacitor, respectively. With the voltage and leakage current at each time step, power consumed due to leakage is extracted and integrated to obtain a nominal energy consumption of 55.97 pJ.

The analog memory and PN junction MRR introduce a bandwidth limitation, which is measured to extract the write time. The nominal write time is measured to be 75.3 ns to 89.4 ns (10-90%) for rise and fall times and 43.8 ns to 63.3 ns for one time constant. Overshoot is observed in the measurement, which is attributed to the lack of termination on chip and inductance in the PCB and wirebonds. The settling time for the circuit given this overshoot is around 3 μs, meaning weights can be updated in 3 μs intervals during training, although pre-emphasis pulse shaping has been shown to effectively deal with such dynamics [1].

3 Supplementary Note 3. Device-Level Comparison of Analog Memory Technologies

Surveyed memory technologies are compared in Supplementary Table 1, focusing on energy consumption, write time, endurance, physical area, and retention time. Among these, DRAM excels in endurance, PCM is optimal in retention time and write time, OAMs have the lowest energy consumption, and floating gate memory requires the least physical space. Area is less critical since photonic devices can amortize memory area within the photonic device. To summarize, each analog memory technology has unique strengths and is suitable for specific applications; no single analog memory technology excels in all aspects.

Figure of Merit	DEOAM (This Work)	DRAM[3]	PCM (E)	OAM	FG	MEMS (O)	MEMS (E)	MO	FE	TC
Endurance (cycles)	8698[1]	10P[2]	2T[3]	2k[4]	10M[5]	30[6]	10G[6]	7[6]	300[6]	30[6]
Retention Time	573.53 μs	64 ms[6]	10 years[7]	1 day[8]	1 year[9]	-	-	-	-	-
Write Energy Consumption (J/bit)	55.97p	3.97p[10]	2.5μ[6]	12.5f[11]	5.4m[12]	10μ[6]	1p[6]	33.3n[6]	10p[6]	7.5p[6]
Write Time	63.3 ns	13.75 ns[13]	500 ps[14]	33.3 ns[11][2]	1.5 ms[12]	125 μs[6][2]	5 μs[6][2]	500 ns[6][2]	500 ns[6][2]	500 ms[6][2]
Area (μm^2)	10000	0.0023[15]	0.001[3]	2[6]	0.00027[16]	400[6]	10000[6]	8000[6]	20000[6]	315[6]

Supplementary Table 1: Key FoMs for state-of-the-art analog memory technologies, which include phase change materials (PCM), optical analog memories (OAM), floating gate (FG) memories, DRAM, micro-electromechanical systems (MEMS), magneto-optic (MO) memories, ferro-electric (FE) memories, and trapped charge (TC) memories. Some analog memory technologies are denoted with electrical (E) or optical (O) for its switching ability.

[1] Write cycles measured until now and can be extended given time for measurement.

[2] Write time is calculated from frequency (t_{wr}=1/(2f) where t_{wr} is the write time in seconds and f is the frequency in Hz).

[3] Reported DRAM characteristics are for isolated DRAM memory chips that are not monolithically integrated with the CMOS computing chip and does not account for leakage in the PN junction MRR and its dependence on optical power.

4 Supplementary Note 4. System-Level Comparison of Analog Memory Technologies

Analog memory technologies from Supplementary Table 1 are used in the weight bank emulation to compare system-level performance metrics, including training energy consumption, training time, and analog memory endurance. These factors are directly impacted by the number of weight updates during training. Supplementary Fig. 2 compares analog memory technologies based on their characteristics from Supplementary Table 1 and the number of weight updates (or writes to analog memory). Each analog memory technology exhibit strengths in different performance aspects. Reliability, for example, is a critical factor because even if an analog memory cell has a latency of 1 ns, its usefulness is limited if it can only endure 1 million writes or 1 ms before it requires recalibration. Therefore, any analog memory in the red zone in Supplementary Fig. 2a is deemed unsuitable. DRAM, PCM, and MEMS (E) demonstrate high endurance against numerous writes, making them superior candidates for reliability. Since DEOAM operates similarly to DRAM and MEMS (E), DEOAM's endurance is expected to reach similar endurance as DRAM and MEMS (E). Regarding energy consumption in Supplementary Fig. 2b, OAMs achieve the lowest energy consumption at 12.5 fJ/bit with MEMS (E) and DRAM following as the next most efficient technologies. In Supplementary Fig. 2c, training time is assumed to be limited by the write time of the analog memory and the number of weight updates. Here, PCMs are the optimal analog memory technology for minimal training time. Since different analog memory technologies excel in specific performance metrics, selecting the most suitable analog memory technology or combinations of technologies for a given neural network application is crucial to optimizing overall system performance [17].

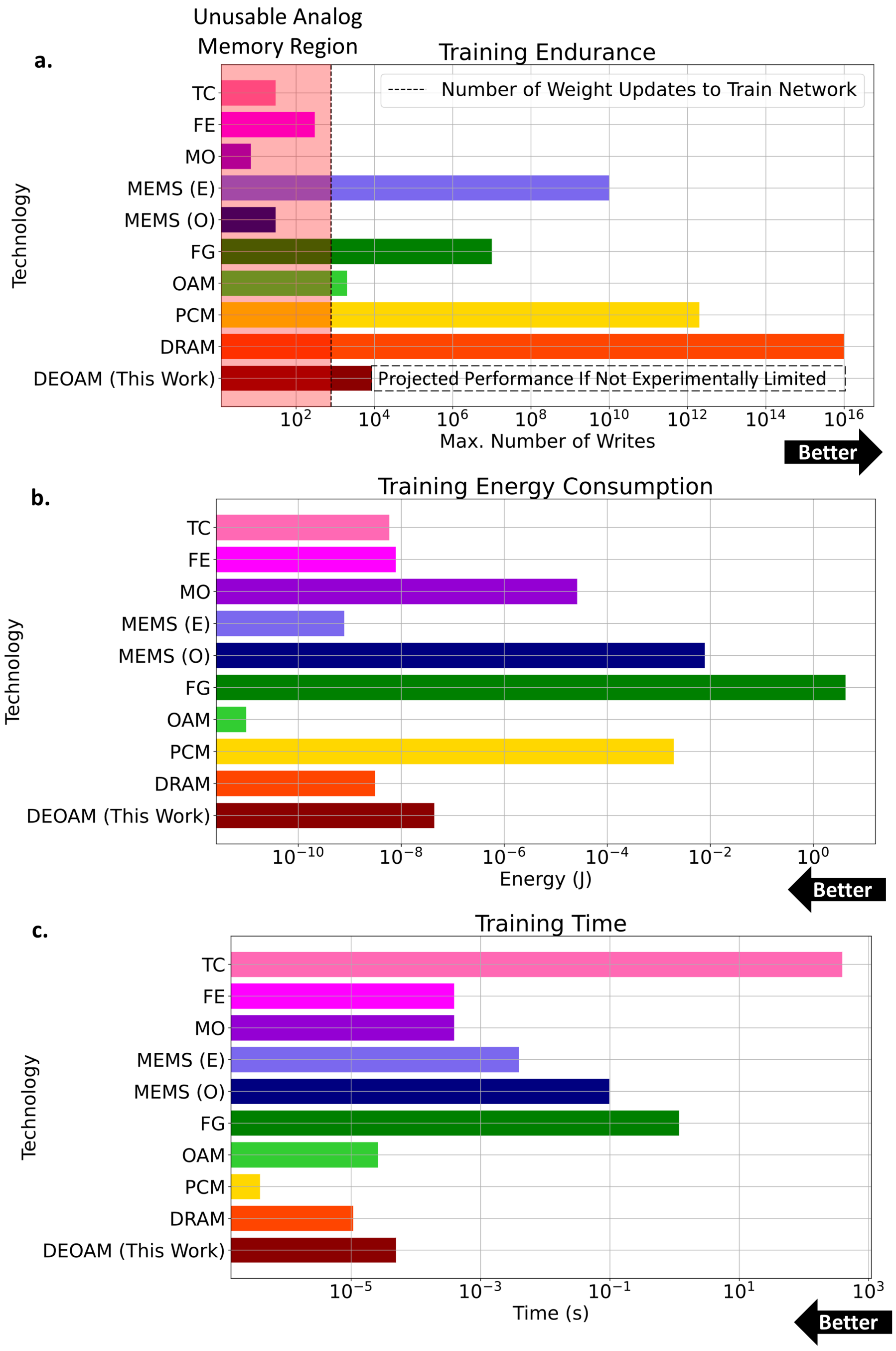

Supplementary Fig. 2: Emulated system comparison of various analog memory technologies and effects on training. a) Any analog memory in the red zone of the training endurance graph will fail or require recalibration before training completes. DRAM is optimal for endurance. b) Energy consumption of analog memories is governed by the write energy. OAMs show the least energy consumption in training. c) Training time for an analog memory is limited by its write time. PCM has the lowest training time.

Different analog memory technologies excel in specific performance metrics but also have certain drawbacks. According to Supplementary Fig. 2c, PCMs demonstrate the fastest training times due to their low write times; however, they consume significant power. On the other hand, OAMs, as shown in Supplementary Fig. 2b,

are the most energy efficient because their write energy per bit is the lowest, but their endurance is marginally sufficient. By leveraging various analog memory technologies to exploit their specific strengths, it is possible to optimize neural network performance [17]. For example, DRAM or DEOAM can be used during training, owing to their predicted high endurance and relatively fast write times. During inference, non-volatile PCMs are suitable since no weight updates occur. While each type of analog memory may excel in one metric, combining different analog memory technologies may complement each other, leading to improved overall system performance.

5 Supplementary Note 5. Effects of Retention Time Constant, Network Latency, and Batch Size on Inference Accuracy

The effects of retention time constant, network latency, and batch size on inference accuracy are shown in Supplementary Fig. 3. When weights are trained without accounting for analog memory retention time and network latency (Supplementary Fig. 3a), inference accuracy degrades at a ratio of analog memory retention time to network latency of about 200. Therefore, extending the ratio of retention time to network latency greater than 200 provides stable and high inference accuracy. Figures 3b-d show inference accuracy when weights are trained to account for analog memory retention time and network latency. Batch sizes of 64, 32, and 16 reveal that inference accuracy degrades at ratios of retention time to network latency of about 100, 40, and 30, respectively. This indicates that training with the effects of analog memory retention time and network latency along with smaller batch sizes relaxes the analog memory retention time constant requirement. One reason is that training with these effects allows the neural network to compensate for these faults. Another reason is that large batch sizes require longer retention times since weights are updated less frequently. Whereas, smaller batch sizes are updated more frequently, which relaxes the analog memory retention time constant requirement.

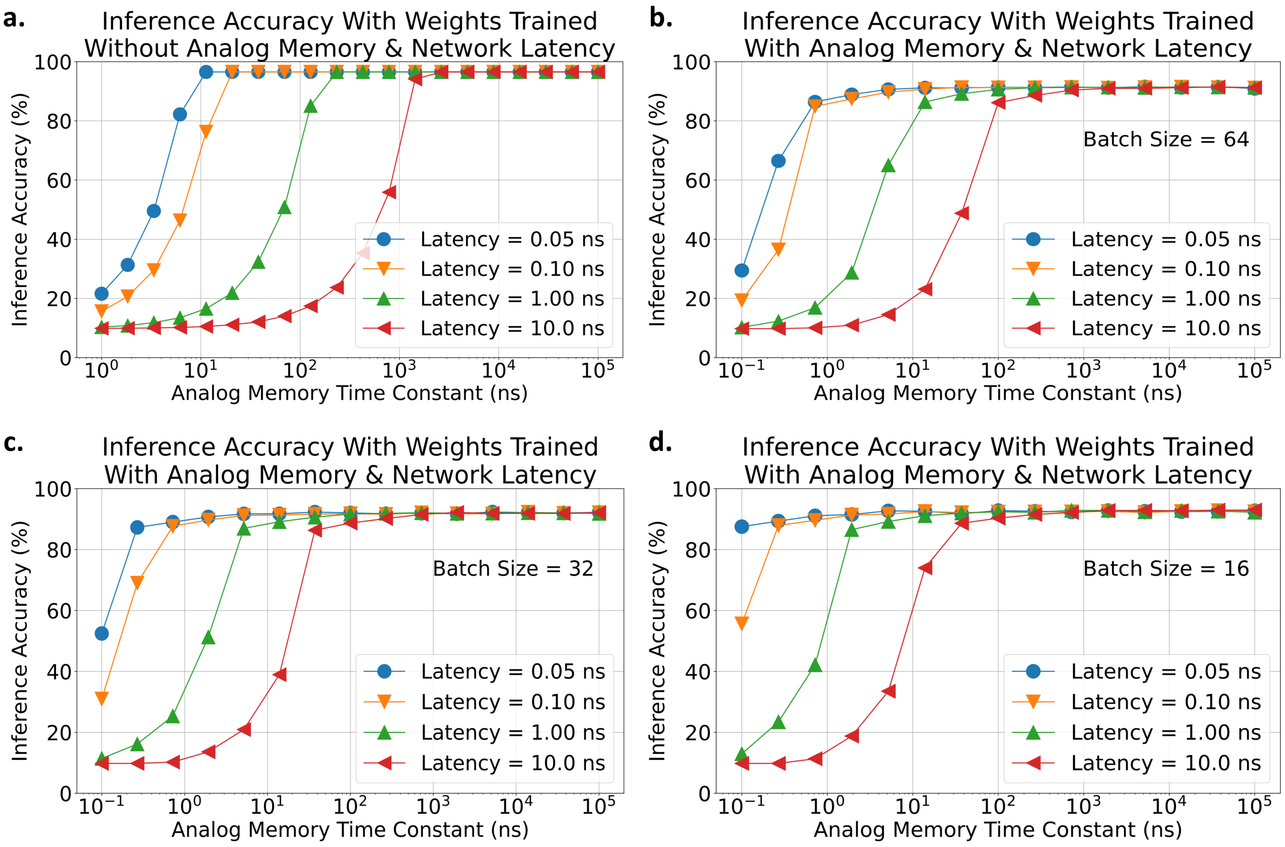

Supplementary Fig. 3: Inference accuracy with varying analog memory time constants, network latencies, and batch sizes. The sweep of network latency and retention time characterizes inference accuracy for weights trained a) without leaky analog memory and network latency and b) with leaky analog memory and network latency. a) A ratio of latency to retention time of 100-300 is sufficient to achieve more than 95% inference accuracy. b) A ratio of latency to retention time of 100 is sufficient to achieve more than 90% inference accuracy. Batch size of b) 64, c) 32, and d) 16 reveal that large batch sizes require longer retention times since weights are updated less frequently. Weights are updated more frequently in smaller batch sizes.

6 Supplementary Note 6. Energy Delay Product

Energy consumption is dependent on the neural network architecture and key devices that consume power, which include lasers, SOAs, DEOAM, PDs, TIAs, input data modulators, and thermal stabilizers. Supplementary Table 2 summarizes the energy consumption of key devices in the neuromorphic photonic circuit. Supplementary Table 2 reveals that optical power delivery via lasers and SOAs and thermal stabilizers consume most of the energy during training followed by DEOAM. Thermal stabilizers using metallic heaters can be replaced by more efficient phase shifters, thereby reducing the energy consumption significantly [18, 19]. Future research for energy efficient neuromorphic photonic processors should, therefore, focus on improving optical power delivery (lasers and SOAs), photonic device stabilization (efficient phase shifters and circuits), and analog memory technology (materials and circuits).

Specification	Laser [20]	SOA [21]	DEOAM (This Work)	PD and TIA [22]	Input Data Modulators [23]	Thermal Stabilizers [24]
Average Power Consumption Per Device (mW)	100 - 1000	300 - 1000	1[1]	37	-	<30
Energy Consumption Per Device (μJ)	5 - 50	15 - 50	0.05	1.85	40E-9 - 1E-6	<1.5
Required Number of Devices	10	50x10 = 500	50x80x10 = 40000	50x10 = 500	80x10 = 800	50x80x10 + 10 = 40010
Total Energy Consumption (μJ)	50 - 500	7500 - 25000	2000	925	32E-6 - 800E-6	<60015

Supplementary Table 2: Training energy consumption analysis for the neuromorphic photonic circuit using DEOAM. Total energy consumption for each device group is calculated from average power consumption, training time (for DEOAM which is 50 μs from Supplementary Fig. 2), and number of devices required for the neural network. Results show that optical power delivery (lasers and SOAs), thermal stabilization, and DEOAM consume the most amount of energy.

[1] Calculated based on nominal write energy and write time.

To analyze the power consumption as the network size scales, we consider an $n \times n$ network comprising n inputs, $n \times n$ weights, and n outputs. In such a configuration, the system includes: n input data modulators, each with a thermal stabilizer, n SOAs, n PD and TIAs, n^2 MRRs each with a DEOAM and thermal stabilizer, and lasers that are assumed to be shared among a maximum of 50×50 MRRs. The total power consumption is then calculated using the device power values listed in Supplementary Table 2 and the following equation:

$$P_{total} = \mathrm{ceil}(\frac{n}{50})P_{laser} + nP_{SOA} + n^2P_{DEOAM} + nP_{PD+TIA} + nP_{input-mod} + (n+n^2)P_{therm} \quad (1)$$

where P_{total} is the total power consumption; ceil is the ceiling function; P_{laser}, P_{SOA}, P_{DEOAM}, $P_{\text{PD+TIA}}$, $P_{\text{input-mod}}$, and P_{therm} are the single device power consumption values for the laser, SOA, DEOAM, PD and TIA, input data modulators, and thermal stabilizers, respectively.

The inference delay path, t_{delay}, starts from the input data drivers and modulators, passes through waveguides, splitters, weight banks, and ends at the PD and TIA. Optical delay includes both the optical path length and the resonance build up time. It is given by:

$$t_{delay} = t_{DRV+MOD} + \frac{n_g}{c}(2nd_{MRR} + FR + L_{splitter}log_2(n)) + t_{PD+TIA} \quad (2)$$

where $t_{\text{DRV+MOD}}$ is the combined propagation delay of the driver and activation modulator (assumed to be data-rate limited, ranging from 10 Gb/s to 200 Gb/s [23]), n_g is the optical group index, c is the speed of light, d_{MRR} is the MRR separation distance, F is the MRR finesse, R is the ring radius, $L_{splitter}$ is the length of a splitter, and t_{PD+TIA} is the combined propagation delay of the PD and TIA. The energy consumed per operation E_{op} is:

$$E_{op} = \frac{P_{total}t_{delay}}{n^2} \quad (3)$$

where there are n^2 multiply-accumulate (MAC) operations in an $n \times n$ network. The energy–delay product (EDP) is then:

$$EDP = E_{op}t_{delay} \quad (4)$$

This EDP can be used to compare different neuromorphic processors as shown in Fig. 4. The EDP projection shown is based on the worse device performance surveyed from Supplementary Table 2, providing a conservative performance estimate. While using the best-in-class devices could yield more optimistic results, integrating all such technologies remains a significant challenge. The projected trend indicates that network sizes with n ranging from 100 to 1000 achieve minimal EDP. For $n > 1000$, EDP increases due to rising energy consumption and delay. Current implementations typically exhibit EDP around 10^{-13} or higher. Although present implementations still have higher EDP, the projections show the potential or substantial improvement if optimal devices can be integrated. Furthermore, the EDP trends serve both as targets for future development and as indicators of architectural scalability. This enables neuromorphic hardware designers to assess the feasibility and suitability of neuromorphic photonic systems for large-scale implementations.

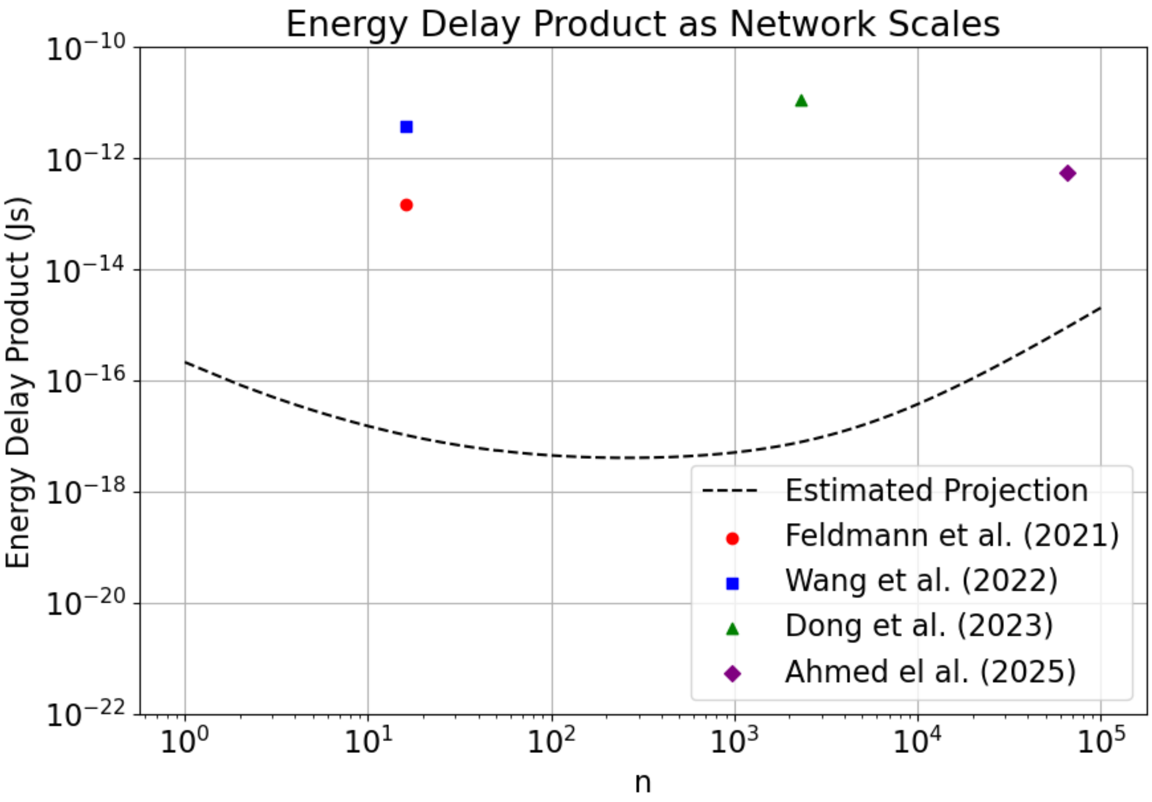

Supplementary Fig. 4: Energy delay product (EDP) as the network scales $n \times n$ (n rows and n columns). The estimated EDP projection uses devices with worse device performance metrics from Supplementary Table 2. Energy delay product is compared to state-of-the-art neuromorphic photonic processors [25].

References

[1] Geis, M., Spector, S., Williamson, R. & Lyszczarz, T. Submicrosecond submilliwatt silicon-on-insulator thermooptic switch. *IEEE Photonics Technology Letters* **16**, 2514–2516 (2004).

[2] Kargar, S. & Nawab, F. Challenges and future directions for energy, latency, and lifetime improvements in NVMs. *Distributed and Parallel Databases* (2022).

[3] Martin-Monier, L. *et al.* Endurance of chalcogenide optical phase change materials: a review. *Optical Materials Express* **12**, 2145–2167 (2022).

[4] Liu, Z. *et al.* Multilevel halide perovskite memristors based on optical & electrical resistive switching effects. *Materials Chemistry and Physics* **288**, 126393 (2022).

[5] Arai, F., Maruyama, T. & Shirota, R. Extended data retention process technology for highly reliable flash EEPROMs of 10/sup 6/ to 10/sup 7/ W/E cycles. *1998 IEEE International Reliability Physics Symposium Proceedings. 36th Annual (Cat. No.98CH36173)* 378–382 (1998).

[6] Youngblood, N., Ríos Ocampo, C. A., Pernice, W. H. P. & Bhaskaran, H. Integrated optical memristors. *Nature Photonics* **17**, 561–572 (2023).

[7] Abdollahramezani, S. *et al.* Tunable nanophotonics enabled by chalcogenide phase-change materials. *Nanophotonics* **9**, 1189–1241 (2020).

[8] Feng, X., Liu, X. & Ang, K.-W. 2D photonic memristor beyond graphene: progress and prospects. *Nanophotonics* **9**, 1579–1599 (2020).

[9] Liu, R., Yang, C.-L. & Wu, W. Optimizing NAND flash-based SSDs via retention relaxation | Proceedings of the 10th USENIX conference on File and Storage Technologies. *FAST'12: Proceedings of the 10th USENIX conference on File and Storage Technologies* (2012).

[10] O'Connor, M. *et al.* Fine-Grained DRAM: Energy-Efficient DRAM for Extreme Bandwidth Systems. *2017 50th Annual IEEE/ACM International Symposium on Microarchitecture (MICRO)* 41–54 (2017). ISSN: 2379-3155.

[11] Koch, U., Hoessbacher, C., Emboras, A. & Leuthold, J. Optical memristive switches. *Journal of Electroceramics* **39**, 239–250 (2017).

[12] Liang, S. *et al.* An Empirical Study of Quad-Level Cell (QLC) NAND Flash SSDs for Big Data Applications. *2019 IEEE International Conference on Big Data (Big Data)* 3676–3685 (2019).

[13] DDR5 SDRAM | JEDEC. URL https://www.jedec.org/standards-documents/docs/jesd79-5b. Accessed on 2023-10-28.

[14] Miller, K. J., Haglund, R. F. & Weiss, S. M. Optical phase change materials in integrated silicon photonic devices: review. *Optical Materials Express* **8**, 2415–2429 (2018).

[15] Choe, J. Memory technology: process and cell architecture. *Optical and EUV Nanolithography XXXVI* **12494**, 1249402 (2023).

[16] Yuh, J. *et al.* A 1-Tb 4b/Cell 4-Plane 162-Layer 3D Flash Memory With a 2.4-Gb/s I/O Speed Interface. *2022 IEEE International Solid- State Circuits Conference (ISSCC)* **65**, 130–132 (2022). ISSN: 2376-8606.

[17] Mukherjee, A., Saurav, K., Nair, P., Shekhar, S. & Lis, M. A Case for Emerging Memories in DNN Accelerators. *2021 Design, Automation & Test in Europe Conference & Exhibition (DATE)* 938–941 (2021). Conference Proceedings.

[18] Taghavi, I. *et al.* Polymer modulators in silicon photonics: review and projections. *Nanophotonics* **11**, 3855–3871 (2022).

[19] Shekhar, S. *et al.* Roadmapping the Next Generation of Silicon Photonics. *Nature Communications* (2024).

[20] Chang, L., Liu, S. & Bowers, J. E. Integrated optical frequency comb technologies. *Nature Photonics* **16**, 95–108 (2022).

[21] Cheung, S., Kawakita, Y., Shang, K. & Yoo, S. J. B. Highly efficient chip-scale III-V/silicon hybrid optical amplifiers. *Optics Express* **23**, 22431–22443 (2015).

[22] Wang, P. *et al.* A Multi-Channel Low-Noise Analog Front End Circuit for Linear LADAR. *IEEE Transactions on Circuits and Systems II: Express Briefs* **67**, 1209–1213 (2020).

[23] Rahim, A. *et al.* Taking silicon photonics modulators to a higher performance level: state-of-the-art and a review of new technologies. *Advanced Photonics* **3**, 024003 (2021).

[24] Liu, S. *et al.* Thermo-optic phase shifters based on silicon-on-insulator platform: state-of-the-art and a review. *Frontiers of Optoelectronics* **15**, 9 (2022).

[25] Ahmed, S. R. *et al.* Universal photonic artificial intelligence acceleration. *Nature* **640**, 368–374 (2025).
